# Blood-based proteomics for schizophrenia: candidate biomarkers for risk assessment and precision psychiatry

**DOI:** 10.3389/fmolb.2026.1882675

**Published:** 2026-09-23

**Authors:** Alexey S. Kononikhin, Anna E. Bugrova, Arina S. Foliforova, Maria I. Indeykina, Polina A. Strelnikova, Natalia V. Zakharova, Anna Y. Morozova, George P. Kostyuk, Evgeny N. Nikolaev

**Affiliations:** 1 The Center for Bio- and Medical Technologies, Skolkovo Institute of Science and Technology, Moscow, Russia; 2 Emanuel Institute of Biochemical Physics, Russian Academy of Science, Moscow, Russia; 3 Moscow Institute of Physics and Technology, Moscow, Russia; 4 Mental-Health Clinic No. 1 named after N.A. Alexeev, Moscow, Russia; 5 I.M. Sechenov First Moscow State Medical University (Sechenov University), Moscow, Russia

**Keywords:** blood biomarkers, clinical high risk, mass spectrometry, olink, precision psychiatry, proteomics, schizophrenia, SOMAscan

## Abstract

Schizophrenia (SCZ) is a severe mental disorder where diagnosis remains subjective, and treatment efficacy is limited by high heterogeneity, particularly for negative and cognitive symptoms. There is an urgent need for objective molecular biomarkers, especially for early diagnosis and for identifying individuals at clinical high risk (CHR) for psychosis. This review analyzes blood proteomic studies in SCZ, CHR states, and related disorders (2019–2025), categorizing them into mass-spectrometry (MS)-based approaches (panoramic and targeted) and affinity-based methods (Olink, SOMAscan). It synthesizes findings from global discovery projects, targeted validation studies, critical risk prediction models, and large-scale Mendelian randomization analyses using UK Biobank data. The field is transitioning from cataloging differentially expressed proteins to identifying causal biomarkers with therapeutic potential (druggability). We propose a consolidated panel of high-priority protein markers, including the ITIH family, CRP, DNER, CTSS, and complement proteins. A critical gap remains in the incomplete coverage of these markers in commercial assays for targeted MS. The future lies in integrating validated proteomic panels with genetic data to enable personalized therapeutic strategies. However, significant hurdles related to reproducibility, cohort heterogeneity, and the lack of standardized validation protocols must be overcome before these biomarkers can be translated into routine clinical practice. Ongoing debates include the choice between MS-based and affinity-based platforms for clinical translation, the interpretation of complement and inflammatory markers as either core pathophysiological drivers or non-specific epiphenomena, and the predictive value of proteomic signatures for psychosis transition. Recent large-scale studies provide a crucial methodological framework, demonstrating the power of rigorous validation and the importance of harmonized, multi-cohort analyses for advancing the field toward clinical implementation.

## 1 Introduction: the need for molecular diagnostics in psychiatry

Schizophrenia (SCZ) is one of the most severe mental disorders, characterized by significant disturbances in reality perception, thinking, emotional regulation, and behavior. According to the World Health Organization, SCZ affects approximately 1 in 300 people worldwide (Institute of Health Metrics and Evaluation). The disease often leads to persistent disability, and patient life expectancy is 10–20 years below the population average, due to high levels of comorbid somatic pathology and mortality across all age categories (Laursen et al., 2014; Charlson et al., 2018).

A key problem is that the diagnosis of SCZ still relies exclusively on clinical criteria (DSM-5/ICD-11). This makes diagnosis subjective and possible only when a full clinical picture emerges, often years after the first symptoms appear (Tandon et al., 2024; Pavlichenko et al., 2020). Particularly important is distinguishing SCZ from bipolar disorder (BD), since these conditions require fundamentally different therapeutic approaches, and misdiagnosis can lead to serious consequences for the patient (Vieta et al., 2018). At the same time, epidemiological evidence indicates that early intervention, especially during the first psychotic episode, critically improves long-term prognosis and social outcomes (Oliver et al., 2018). This has spurred the development of specialized early intervention services, such as “First-Episode Psychosis Clinics,” and intensified the search for biomarkers to identify individuals at clinical high risk (CHR) for psychosis—a state characterized by subthreshold psychotic symptoms and a significant decline in functioning, which confers a greatly increased risk of developing a full psychotic disorder in the near future.

Current antipsychotic medications, mainly targeting dopamine D2 receptors, effectively control positive symptoms (hallucinations, delusions) but have limited impact on negative symptoms (apathy, anhedonia, social isolation) and cognitive impairments, which most significantly determine patient quality of life and functional outcomes (Lu et al., 2026). Moreover, treatment response is highly heterogeneous: up to 30% of patients demonstrate treatment resistance, and side effects (metabolic disturbances, extrapyramidal disorders, hyperprolactinemia) significantly reduce compliance (Lu et al., 2026). Failures of several clinical trials of highly selective compounds (TAAR1 agonists, GlyT-1 inhibitors, glutamate receptor modulators) emphasize that a single-target approach based on a single pathogenetic hypothesis is insufficient for such a heterogeneous disease as SCZ (Lu et al., 2026). This creates an urgent need for biomarkers that could stratify patients into biological subtypes, predict therapy response, and identify new therapeutic targets.

The effectiveness of early intervention clinics could be greatly enhanced by the availability of objective molecular tests, not only for early diagnosis of SCZ but also for its differential diagnosis from other mental disorders with similar clinical presentations in the early stages. Recent large-scale genomic studies, such as the cross-disorder GWAS by Grotzinger et al. (2025) mapping the genetic landscape across 14 psychiatric disorders, have revealed a complex architecture where disorders like schizophrenia and bipolar disorder share a substantial proportion of their genetic risk, while also identifying factor-specific biological pathways, such as enrichment of excitatory neuron biology for the schizophrenia/bipolar factor. These findings underscore that diagnostic boundaries based solely on clinical criteria do not align neatly with underlying biology, further emphasizing the need for objective molecular tools—like blood proteomics—that can capture both shared and disorder-specific pathophysiological signatures to aid in differential diagnosis and patient stratification.

A promising direction for searching diagnostic and therapeutic biomarkers is omics research and the search for panels of molecular markers in blood. Compelling examples of successful use of molecular markers are demonstrated, in particular, by lipid studies. Thus, in the work by Tkachev et al. (2023) on a large international cohort (over 1,500 samples), a reproducible profile of changes in 77 lipids in blood plasma in SCZ was identified, which with high accuracy (AUC 0.86–0.95) distinguished patients from healthy controls. Notably, this lipid signal largely coincided in BD and major depressive disorder (MDD), indicating common molecular patterns associated with severe mental disorders. Another large study within the PsyCourse Study expanded these data, showing a connection between plasma lipid profile and cognitive functions. Analysis of 364 lipids from 16 classes in 623 participants (patients with SCZ, BD, and healthy controls) revealed that levels of three types of phosphatidylethanolamines (plasmalogens) and one ceramide negatively correlated with results of the Digit-Symbol test (information processing speed) (Oraki Kohshour et al., 2025). Moreover, enrichment analysis confirmed the significance of the phosphatidylethanolamine class for this cognitive characteristic, opening potential for developing objective methods for assessing cognitive impairment—a key and difficult-to-treat symptom (Oraki Kohshour et al., 2025).

However, lipid changes reflect only one aspect of the complex pathophysiology of mental disorders, whereas proteins play a key role in implementing genetic information and regulating cellular processes. Proteins are the direct effectors of signaling pathways, mediators of immune reactions, and targets for most medications. Previous proteomic studies have already shown that about 200 blood proteins have significant changes in groups of patients with SCZ, and about 100 were associated with BD (Comes et al., 2018). Most identified potential biomarkers belong to cascades of the immune system and lipid metabolism (Comes et al., 2018; Rosenblat and McIntyre, 2016).

The application of blood proteomics to study CHR individuals is a particularly important and promising direction. Identifying blood-based biomarkers that can predict which CHR individuals will ultimately transition to psychosis could revolutionize early intervention, allowing for targeted and potentially preventative treatments. Early studies in this area, such as the work by Mongan et al. (2020), have shown great promise, suggesting that proteomic data can indeed predict transition with high accuracy in smaller cohorts. Subsequent large-scale validation efforts, by the same group (Byrne et al., 2024), represent a critical next step, applying cutting-edge mass spectrometry to rigorously test initial findings in larger, more representative populations. These validation studies often leverage data from large, well-characterized consortia, such as the North American Prodrome Longitudinal Study (NAPLS-3), which was specifically designed to collect longitudinal clinical and biomarker data from CHR individuals to elucidate the mechanisms of psychosis onset (Addington et al., 2020; Laskaris et al., 2017).

Modern proteomics technologies based on mass spectrometry (panoramic or targeted) and affinity methods (Olink, SOMAscan), both with its advantages and limitations, provide a powerful toolkit for biomarker discovery and validation. Mass spectrometry allows unbiased panoramic search and accurate quantitative analysis using isotopically labeled standards (Carr et al., 2014), while affinity platforms provide high throughput and sensitivity necessary for large-scale population studies. A prime illustration of such platform capabilities is the work by Álvez et al. (2025), which established a comprehensive pan-disease atlas of the circulating proteome through the large-scale analysis of 5,000 proteins in 8,000 individuals.

However, despite the growing flow of data, the field of proteomics of mental disorders is still at the stage of searching for and verifying candidate markers. A consensus biomarker panel ready for implementation in clinical practice has not yet been established. The presented facts demonstrate the high relevance of continuing proteomic research in psychiatry and the need to systematize accumulated knowledge.

The aim of this review is to analyze the current state of blood proteomic research in SCZ, CHR states, and other mental disorders performed by mass spectrometry methods and on the Olink and SOMAscan platforms, as well as to identify the main methodological gaps and substantiate directions for future research. Special emphasis is placed on the importance of rigorous validation, as exemplified by recent large-scale studies, and on the prospects of integrating proteomic data with the concept of “druggability” for moving from empirical treatment selection toward precision medicine based on the individual molecular profile of the patient.

To ensure comprehensive and transparent coverage of the available literature, we performed a systematic search in PubMed, Web of Science, and Scopus databases for articles published between January 2019 and June 2025. The following search terms were used: (“schizophrenia” OR “psychosis” OR “clinical high risk” OR “first-episode psychosis”) AND (“proteomics” OR “mass spectrometry” OR “Olink” OR “SOMAscan” OR “MRM” OR “DIA” OR “DDA”) AND (“plasma” OR “serum” OR “blood” OR “biomarker”). Additional relevant studies were identified by manual screening of reference lists of included articles and key reviews. Studies were included if they (1) reported original research on blood-based proteomics in schizophrenia, clinical high-risk states, or related psychiatric disorders; (2) used mass spectrometry-based (panoramic or targeted) or affinity-based (Olink, SOMAscan) platforms; and (3) were published in peer-reviewed English-language journals. We excluded studies performed on cerebrospinal fluid, post-mortem brain tissue, or non-human models, as well as studies with sample sizes of fewer than 20 participants per group. Given the narrative nature of this review, we prioritized studies with large cohort sizes, multi-center validation, and those that integrated proteomic data with genetic or clinical information to highlight key advances and methodological gaps in the field.

## 2 Mass spectrometry in blood proteomics for schizophrenia

MS-based studies can be divided into panoramic (discovery) studies and targeted studies using multiple reaction monitoring (MRM). A summary of key MS studies, with integrated information on methods and instruments, is provided in Table 1.

**TABLE 1:** Blood proteomic studies in schizophrenia and clinical high risk.

Study	Platform and sample type	Population	Proteins (analyzed/significant)	Representative potential markers (top candidates)	Key findings
Mass spectrometry					
(Shen et al., 2024)	LC-MS/MS (4D-DIA, timsTOF Pro); plasma	SCZ (40), HC (40)	405/79	Top markers from ML model: PFN1, GAPDH, ACTBL2, LYZ, ORM2, PON1, CAT	Identified 79 differentially expressed proteins; proposed ML-based diagnostic model with AUC = 0.972
(Petrovskiy et al., 2024)	LC-MS/MS (DDA, Orbitrap fusion); depleted plasma	SCZ (48), HC (50)	907/18	Synaptic plasticity-related: ZNF48, SAA2, ITIH5, DLG4, EFNB3, FLT1	Identified 18 proteins linked to synaptic plasticity and neurodevelopment
Kopylov et al., 2023)	LC-MS/MS (Orbitrap fusion); plasma	SCZ (77), HC (61)	248/20	Lipid metabolism and complement: IGFALS, ORM1, AHSG, ITIH4, APOC3	Identified 20 proteins related to lipid metabolism, complement, and transport; integrated with metabolomics and GWAS
(Campeau et al., 2022)	LC-MS/MS (Orbitrap fusion); plasma, high pH LC fractionation	SCZ (54), HC (51)	742/45	Accelerated aging cluster: C3, CST3, CRP, APOE, C5, IGFBP3	Confirmed accelerated aging hypothesis; identified protein cluster linked to somatic comorbidities in young patients
(Mongan et al., 2020)	LC-MS/MS (Q-exactive, DDA); plasma	CHR converters (49), CHR non-converters (84)	345/35 (FDR <0.05)	Top 10 predictors in SVM model: A2M, IGHM, PLG, CLU, C6, CPN2, PROS1, GC, C1S, TTR	Developed SVM model with AUC = 0.95; 10-protein model AUC = 0.92 for predicting transition to psychosis
(Byrne et al., 2024)	LC-MS/MS (timsTOF Pro, label-free); plasma	CHR converters (154), CHR non-converters (600)	99/0 (after correction)	Nominal associations (uncorrected): C8B, C4B, C5, LRG1	Large-scale validation study (n = 754); 10-protein panel did not replicate (C-statistic ∼0.51); provided insights into generalizability
(Smirnova et al., 2019)	LC-MS/MS (DDA, Orbitrap); depleted serum	SCZ (33), BD (23), HC (24)	∼1,600/27 (SCZ) + 18 (BD)	SCZ-specific (validated): ANKRD12, SERPINA3, APOE, F12, PROS	Identified 27 SCZ-specific proteins; validated ANKRD12 by ELISA in independent cohort
(Shin et al., 2024)	MRM-MS (triple quadrupole, QQQ); plasma	SCZ (23), BD (47), MDD (35), HC (27)	452 (targeted)/13 (in final panel)	Multi-protein panel for differential diagnosis: GPR37, SERPINA6 (CBG), SAA1, SERPIND1 (HEP2), SERPING1 (IC1), TF, and PROS1	Developed multi-protein models: SCZ vs. HC AUC = 0.955; SCZ vs. BD AUC = 0.948
Affinity platforms					
(Eren et al., 2023)	Olink (inflammation panel, 92-plex); plasma and CSF	FEP (43 later diagnosed with SCZ, 34 other psychoses), HC (56)	92/15 (plasma) and 47/1 (CSF)	Inflammatory markers: AXIN1, STAMBP, SIRT2, CASP-8, IL-10RB, ST1A1, CD5	Identified 15 systemic inflammatory proteins elevated in FEP vs. HC; distinct signature correlated with PANSS severity. Demonstrated strong blood-CSF correlation for the CD5 marker
(Malmqvist et al., 2025)	Olink (cardiovascular panel, 92-plex); plasma	FEP (42 with confirmed SCZ, 30 without SCZ), HC (54)	92/16	Cardiovascular risk markers: PCSK9, MMP-12, PLA2G7, TNFRSF11B	Identified 16 proteins linking SCZ to cardiovascular risk; suggested shared pathophysiology
(Engelke et al., 2022)	SOMAscan (1.3K panel, 1,301-plex); plasma	SСZ/BD on APs (66), SСZ/BD off APs (29), HC (102)	1,301/58 (FDR <0.05)	Disease state markers: NTRK2, CNTN1, ROBO2, PLXNC1 Medication markers: NCAM1, TNFRSF17 Diagnostic model: PRL, MRC2 (AUC = 0.93)	Distinguished disease-related state markers from medication-specific effects; AP-free patients showed downregulation of neurodevelopmental proteins
(Zhang et al., 2025)	Olink (explore 3072 panel); plasma	SCZ (up to 53,386), BD (up to 41,917), MDD (up to 170,756), 9 other disorders; UK Biobank + PGC	∼3,000/53 causal (cross-disorder)	Causal proteins with druggability potential: ITIH3 (clozapine target), BTN2A1, BTN3A2, FES, SERPINA6	MR identified 53 potentially causal proteins across 12 disorders; druggability analysis highlighted ITIH3 as clozapine target
(Chen et al., 2025)	Olink (inflammation panel, 92-plex); plasma	SCZ (up to 53,386), BD (up to 41,917), MDD (up to 170,756); UK Biobank + PGC	95/3 causal (SCZ), 3 (BD), 4 (MDD)	High-priority causal targets: DNER (SCZ risk); TNFSF12 and FGF23 (MDD protective)	MR and colocalization identified DNER as high-priority causal target for SCZ
(Bhattacharyya et al., 2025)	Olink (explore 3072 panel) + SOMAscan (7K panel); plasma	SCZ (up to 76,755), BD (up to 41,917), MDD (up to 170,756); UK Biobank + PGC	Olink (∼3,000) + SOMAscan (∼7,000)/91 causal (46 novel)	Novel causal proteins: ITIH1, ITIH3, ITIH4, CTSS, complement components (C3, C4, C5, C8B)	Largest MR study to date; identified 91 proteins (46 novel) for SCZ, BD, MDD, and cognition

Abbreviations: SCZ, schizophrenia; BD, bipolar disorder; MDD, major depressive disorder; CHR, clinical high risk for psychosis; FEP, first-episode psychosis; HC, healthy controls; APs, antipsychotics; PGC, psychiatric genomics consortium; MR, mendelian randomization; MS, mass spectrometry; LC-MS/MS, liquid chromatography-tandem mass spectrometry; DDA, data-dependent acquisition; DIA, data-independent acquisition; MRM, multiple reaction monitoring; ML, machine learning; AUC, area under the curve; FDR, false discovery rate; SVM, support vector machine.

A meta-analysis by Rodrigues et al. (2022) of 19 MS studies identified 197 biomarkers, however, overlap between plasma and serum was only 20 proteins, highlighting a key challenge of reproducibility and sample-type dependence. Smirnova et al. (2019) used serum depletion on an Orbitrap instrument to directly compare SCZ and BD, identifying 27 SCZ-specific proteins and validating ANKRD12 by ELISA (Smirnova et al., 2019). Other groups identified different sets of SCZ-specific proteins using Orbitrap Fusion system: Petrovskiy et al. (2024) employed plasma depletion (top 14 proteins) to achieve extended proteome coverage, identifying 907 proteins with 18 linked to synaptic plasticity. In contrast, Kopylov et al. (2023) analyzed non-depleted plasma from a larger cohort (77 SCZ, 61 HC), identifying 248 proteins with 20 related to lipid metabolism and complement. This difference in proteomic depth (907 vs. 248 proteins) highlights the trade-off between broader coverage achieved by depletion strategies and the reduced sample manipulation and potential biases associated with non-depleted approaches. Shen et al. (2024) employed a timsTOF Pro with 4D-DIA technology to identify 79 proteins and proposed a machine learning-based diagnostic model (AUC = 0.972).

Landmark multi-omics projects like Campeau et al. (2022) used an Orbitrap Fusion to confirm the accelerated aging hypothesis in SCZ, identifying a protein cluster (C3, CST3, CRP, ApoE) linked to somatic comorbidities and elevated in young patients. The work by Shin et al. (2024) has made the most significant progress towards clinical validation. Using MRM on a triple quadrupole instrument with a 453-plex panel, they developed multi-protein models for differential diagnosis, achieving high accuracy in distinguishing SCZ from HC (AUC = 0.955) and SCZ from BD (AUC = 0.948). The panel included CRYM, CBG, SAA1, and HEP2. A significant translational bottleneck is that key markers like CRYM are absent from major commercial MRM panels (Gaither et al., 2020; Strelnikova et al., 2025; Lesur et al., 2023).

The study by Mongan et al. (2020) provided early, promising evidence that proteomic biomarkers could aid in predicting transition to psychosis in CHR individuals. Using a discovery-based approach on a nested case-control sample from the EU-GEI consortium (49 who progressed to psychosis, 84 who did not), they developed a support vector machine model incorporating clinical and proteomic data that demonstrated excellent predictive performance (AUC = 0.95). A more parsimonious model based on just the 10 most predictive proteins (including A2M, IGHM, PLG, CLU, and several complement components) was internally validated in a held-out test set, retaining high accuracy (AUC = 0.92). Functional analysis pointed to dysregulation in the complement and coagulation cascade as a key feature associated with transition.

A pivotal follow-up study by the same group (Byrne et al., 2024) exemplifies the crucial next step in biomarker development: large-scale, rigorous validation using cutting-edge technology. This work leveraged a much larger multi-cohort sample of 754 CHR individuals—including participants from the NAPLS-2, NAPLS-3, and NEURAPRO studies—and state-of-the-art timsTOF Pro mass spectrometry to test the generalizability of the previous findings. While the specific 10-protein panel did not show high predictive accuracy in this expanded cohort (C-statistic ∼0.51), the study was immensely valuable. It demonstrated the power of well-powered validation studies to provide a more accurate estimate of true effect sizes and identified nominally significant associations for complement proteins (C8B, C4B, C5) and LRG1, offering new, more robust candidate markers for future investigation. This work sets a new standard for the field, showing how proteomic research must evolve from initial discovery to large-scale, multi-cohort validation.

## 3 Affinity-based proteomics: from targeted panels to population studies

Affinity-based proteomic platforms, including Olink and SOMAscan, have become leaders in the field due to their high throughput, specificity, and multiplexing capability. These technologies enable simultaneous quantification of hundreds to thousands of proteins with high sensitivity, making them particularly suitable for large-scale population studies and for testing specific biological hypotheses using specialized panels. While Olink utilizes Proximity Extension Assay (PEA) technology, SOMAscan employs Slow Off-rate Modified Aptamers (SOMAmers) that bind to protein targets with high specificity and affinity. Both approaches have been successfully integrated with genetic data to move the field from association to causation. A summary of key affinity-based studies is provided in Table 1.

Eren et al. (2023) used an Olink inflammation panel in a cohort of 77 first-episode psychosis (FEP) patients (43 later converted to SCZ) and 56 HC to identify 15 elevated proteins. Malmqvist et al. (2025) used an Olink cardiovascular panel on 72 FEP patients (42 with confirmed SCZ) and 54 HC, finding 16 proteins elevated in SCZ, linking the disorder to cardiovascular risk.

Engelke et al. (2022) employed the SOMAscan platform to quantify 1,301 proteins in plasma from patients with SZ and BD, comparing those maintained on antipsychotics (APs) to AP-free patients and healthy controls. This study design allowed for disentangling disease-related protein changes from medication effects. The authors identified proteins involved in nervous system differentiation (NTRK2, CNTN1, ROBO2, PLXNC1) that were downregulated in AP-free patients but normalized in those on APs, suggesting these may represent state markers. Conversely, a cluster of immune-related proteins (NCAM1, TNFRSF17) was normal in AP-free patients but downregulated in medicated patients, indicating medication-specific effects. Using random forest modeling, the study achieved excellent discrimination of SZ from HC using only two proteins (PRL and MRC2, AUC = 0.93), while BD prediction required more markers and yielded lower accuracy (AUC = 0.91 with ten proteins). This study provides a critical framework for interpreting proteomic data by demonstrating that some changes (e.g., downregulation of NTRK2, CNTN1, ROBO2) are state-dependent disease markers that normalize with treatment, while others (e.g., NCAM1, TNFRSF17) represent a direct pharmacological footprint of antipsychotics. This distinction is essential; without accounting for medication status, a significant portion of the proteomic signal could be misattributed to the pathophysiology of schizophrenia itself, potentially leading to the selection of non-specific biomarkers.

Integrating proteomics with genetics via Mendelian Randomization using large-scale UK Biobank Olink data has moved the field from association to causation. Zhang et al. (2025) using the Olink Explore 3072 panel identified 53 potentially causal proteins across 12 psychiatric disorders, with druggability analysis highlighting ITIH3 as a target for clozapine. Chen et al. (2025) analyzed 95 inflammatory proteins (Olink Inflammation panel, 92-plex), finding colocalization of genetic variants for DNER and SCZ, making DNER a high-priority causal target. Bhattacharyya et al., (2025) conducted the largest MR study to date, combining Olink Explore 3072 and SOMAscan 7K panel data to identify 91 proteins, including 46 novel ones for SCZ, BD, MDD, and cognition.

## 4 Convergence of mass spectrometry and affinity-based proteomics in schizophrenia biomarker discovery

MS and affinity platforms (Olink and SOMAscan) differ fundamentally in their principles, with the former relying on direct peptide detection and the latter employing antibody- or aptamer-based capture. MS, particularly in its targeted configuration (e.g., MRM), offers unparalleled quantitative accuracy and specificity, enabling absolute quantification of proteins using isotopically labeled internal standards (Carr et al., 2014; Gaither et al., 2020; Lesur et al., 2023). This makes targeted MS the “gold standard” for validation of candidate biomarkers (Strelnikova et al., 2025; Ignjatovic et al., 2019). Panoramic MS approaches (DDA, DIA) allow for unbiased discovery of novel proteins without prior selection, though they typically have lower throughput and greater challenges in standardization across studies (Rodrigues et al., 2022; Ignjatovic et al., 2019; Geyer et al., 2019; Greco et al., 2017). Affinity platforms like Olink and SOMAscan, in contrast, offer high throughput and multiplexing capability, allowing quantification of hundreds to thousands of proteins in large cohorts with high sensitivity (Álvez et al., 2025; Engelke et al., 2022). These platforms are particularly well-suited for large-scale population studies and have been successfully integrated with genetic data to perform Mendelian randomization, establishing causal relationships between circulating proteins and disease (Zhang et al., 2025; Chen et al., 2025; Bhattacharyya et al., 2025). However, they are limited to predefined protein panels and may be subject to antibody cross-reactivity or aptamer binding specificity issues.

Both approaches are widely used in research on schizophrenia and related disorders, providing complementary data for a more comprehensive understanding of the circulating proteome. Despite their methodological differences, there is notable convergence between MS and affinity platforms for several key protein classes and pathways, as summarized in Table 2 (Platform Availability and Consistency column). This cross-platform validation strengthens the evidence for these proteins as robust candidate biomarkers and highlights core pathophysiological mechanisms in schizophrenia.

**TABLE 2:** Consolidated high-priority candidate protein markers in schizophrenia.

Protein	Disease association	Biological role and clinical potential	Validation type and evidence strength	Platform availability and consistency
Acute phase and Reelin pathway				
ITIH1, ITIH4	SCZ, BD	Role: acute phase response, Reelin pathway stabilization. Potential: diagnosis; cognitive impairment assessment	MR (Olink + SOMAscan) (Bhattacharyya et al., 2025); colocalization (Kopylov et al., 2023; Zhang et al., 2025); MS validation in SCZ plasma (Kopylov et al., 2023)	High (MS + Affinity + Genetics). Panels: BAK270(+), PQ500(+), Olink(+), SOMAscan(+)
ITIH3	SCZ, BD, cognition	Role: acute phase response, Reelin pathway. Potential: therapeutic target (clozapine interaction); differential diagnosis	MR (Bhattacharyya et al., 2025); colocalization (Zhang et al., 2025); druggability: clozapine target (Zhang et al., 2025); MS panel for differential diagnosis (Shin et al., 2024)	High (MS + Affinity + Genetics). Panels: BAK270(−), PQ500(+), Olink(+), SOMAscan(+)
Inflammatory markers				
CRP	SCZ (protective)	Role: systemic inflammation marker. Potential: risk assessment; monitoring inflammation; patient stratification for anti-inflammatory therapy	MR (causal, protective) (Bhattacharyya et al., 2025); meta-analysis of MS studies (Campeau et al., 2022); replicated across multiple cohorts	High (MS + Affinity + Genetics). Panels: BAK270(+), PQ500(+), Olink(+), SOMAscan(+)
AHSG, SERPINA6	SCZ, BD	Role: transport protein; corticosteroid-binding globulin. Potential: Differential diagnosis; monitoring stress response	MR (causal) (Bhattacharyya et al., 2025); MS validation (Kopylov et al., 2023; Campeau et al., 2022); affinity replication (Engelke et al., 2022)	High (MS + Affinity). Panels: BAK270(−), PQ500(+), Olink(+), SOMAscan(+)
Complement system				
C3, C4A, C4B, C5, C8B	SCZ, CHR, aging	Role: Complement cascade activation; synaptic pruning; neuroinflammation. Potential: Risk assessment; monitoring disease activity; therapeutic target (complement inhibition)	Genetics: C4 structural variation confers risk (Sek et al., 2016); transcriptomics: C4A overexpression (Fromer et al., 2016); Proteomics: elevated in SCZ (Byrne et al., 2024; Campeau et al., 2022); MR causal (Bhattacharyya et al., 2025); CHR: C4B, C5 associated with transition (Byrne et al., 2024)	High (Genetics + Transcriptomics + MS + Affinity). Panels: BAK270(+ for C3,C5); PQ500(+ for C3,C5); Olink(+); SOMAscan(+)
Differential diagnosis panel (MRM-validated)				
CRYM, CBG, SAA1, HEP2, IC1, TRFE, PROS	SCZ, BD, MDD	Role: thyroid hormone binding; transport; acute phase; protease inhibition. Potential: differential diagnosis (SCZ vs. BD vs. MDD) — most clinically validated panel to date	MRM-MS model (453-plex targeted panel) (Shin et al., 2024); SCZ vs. HC AUC = 0.955; SCZ vs. BD AUC = 0.948; replicated in independent test set	Moderate (MS only, MRM-validated). Panels: BAK270(+), PQ500(+), Olink(+), SOMAscan(−)
Disease state markers (AP-free vs. AP-treated)				
NTRK2, CNTN1, ROBO2, PLXNC1	SCZ (state-dependent)	Role: neuronal differentiation; axon guidance; synaptic plasticity. Potential: monitoring disease activity; distinguishing state from trait; treatment response monitoring	SOMAscan discovery (Engelke et al., 2022); Downregulated in AP-free patients; normalized with AP treatment (state marker); GWAS support (Lee et al., 2013; Lam et al., 2019; Coleman et al., 2020; Wu et al., 2020)	Moderate (Affinity + GWAS support). Panels: BAK270(−), PQ500(−), Olink(−), SOMAscan(+)
Medication effect markers				
NCAM1, TNFRSF17	SCZ (medication-related)	Role: immune function; cell adhesion. Potential: monitoring antipsychotic medication effects; distinguishing drug effects from disease biology	SOMAscan discovery (Engelke et al., 2022); downregulated by APs independently of disease (medication signature)	Moderate (Affinity only). Panels: BAK270(−), PQ500(−), Olink(−), SOMAscan(+)
Other MR-identified causal proteins				
DNER	SCZ (risk)	Role: neurodevelopmental receptor; neuronal differentiation. Potential: therapeutic target (novel class); early diagnosis	MR (causal) (Chen et al., 2025); colocalization with SCZ GWAS loci (Chen et al., 2025); high-priority target	Moderate (Affinity + Genetics). Panels: BAK270(−), PQ500(−), Olink(−), SOMAscan(−)
CTSS	SCZ	Role: immune regulation (cathepsin S); antigen presentation. Potential: causal target; diagnosis	MR (combined Olink + SOMAscan) (Bhattacharyya et al., 2025); novel causal protein	Moderate (Affinity + Genetics). Panels: BAK270(−), PQ500(−), Olink(−), SOMAscan(−)

Abbreviations: SCZ, schizophrenia; BD, bipolar disorder; MDD, major depressive disorder; CHR, clinical high risk for psychosis; HC, healthy controls; AP, antipsychotics; MR, mendelian randomization; MS, mass spectrometry; MRM, multiple reaction monitoring; AUC, area under the curve; BAK270, PeptiQuant™ Plus Human Plasma Kit (270 proteins, MRM-based); PQ500, Biognosys kit (MRM-based); Olink, Olink Explore platform (PEA, technology); SOMAscan, SOMAscan platform (aptamer-based); +, protein is detectable or included in the standard panel; –, not covered or not available in the standard commercial panel. Platform Consistency: High, validated across at least two independent platforms with consistent direction of effect; Moderate, validated within a single platform or with limited cross-platform data, but with strong supporting evidence (e.g., GWAS, MR).

The comparison reveals that MS and affinity platforms provide complementary data, with affinity platforms facilitating causal inference via Mendelian randomization (Zhang et al., 2025; Chen et al., 2025; Bhattacharyya et al., 2025) and specialized studies allowing for the separation of disease from medication effects (Engelke et al., 2022). While targeted MS (MRM) offers high accuracy for clinical diagnosis, a translational gap exists, as key causal markers from large-scale studies are not currently covered by commercial MRM panels (Gaither et al., 2020).

The convergence of findings across platforms for complement and ITIH family proteins, combined with genetic evidence from GWAS (Grotzinger et al., 2025), provides strong support for these pathways as central to schizophrenia pathophysiology, with the complement system emerging as a particularly unifying theme implicated at the genetic (C4), transcriptional (C4A expression), and proteomic (C3, C4, C5, C8B) levels across both MS and affinity platforms. To advance these insights, future studies should prioritize multi-platform validation of candidate markers using both MS and affinity methods in the same cohorts, as well as the expansion of commercial MRM panels to include markers identified from large-scale MR studies. Furthermore, it is essential to integrate medication status into study designs, following the model of Engelke et al. (2022), and to focus on the harmonization of protocols across centers to improve global reproducibility (Ignjatovic et al., 2019; Geyer et al., 2019; Greco et al., 2017).

## 5 Candidate blood protein markers for diagnosis and personalized therapy

Integrating the wealth of proteomic findings from both MS-based and affinity-based studies with the current understanding of schizophrenia pathogenesis (Lu et al., 2026) allows for the prioritization of markers with the greatest clinical potential. Among the consolidated candidates in Table 2, several stand out for their specific clinical promise.

Markers of systemic inflammation such as CRP, IL-6, and TNF-α, while not specific to SCZ, offer tools for monitoring disease activity and patient stratification for anti-inflammatory interventions. For differential diagnosis, panels including CRYM, CBG, and SAA1 have shown high accuracy in distinguishing SCZ from BD and MDD in MRM-based models (Shin et al., 2024). From a therapeutic perspective, ITIH3 stands out as a particularly promising target, given its genetic association with SCZ and its identification as a target of clozapine (Zhang et al., 2025). Furthermore, novel proteins like DNER and CTSS, identified through Mendelian randomization, represent entirely new classes of potential drug targets linked to neurodevelopment and immune regulation, respectively (Chen et al., 2025; Bhattacharyya et al., 2025).

A particularly compelling and well-validated class of markers are proteins of the complement system, which have been consistently implicated in SCZ pathogenesis through genetic, transcriptomic, and proteomic studies (Woo et al., 2020; Nimgaonkar et al., 2017; Heurich et al., 2022). The complement system represents a unifying theme in schizophrenia pathophysiology, linking genetic risk, transcriptomic changes, and proteomic findings. The landmark finding that structural variation in the complement C4 gene confers genetic risk for schizophrenia (Sek et al., 2016) has been mechanistically linked to increased C4A expression in post-mortem brains (Fromer et al., 2016) and, more recently, to increased levels of the C4 activation product (C4-ana) in the plasma of patients with early-course schizophrenia (Kalinowski et al., 2021). This is hypothesized to lead to excessive synaptic pruning during critical developmental periods, particularly in the prefrontal cortex (Donohoe et al., 2013). Our review highlights that this genetic vulnerability translates into a measurable proteomic signature in the blood, with studies consistently reporting elevated levels of C3, C5, C8B, and the C4 activation product (C4-ana) (Byrne et al., 2024; Campeau et al., 2022; Kalinowski et al., 2021>). The detection of C4-ana in plasma (Kalinowski et al., 2021) is particularly significant, as it provides a direct link between genetic risk (C4A copy number) and a functional protein product in the blood, and also suggests a potential mechanism—increased blood-brain barrier permeability—by which complement dysregulation could contribute to pathology. The association of complement markers with psychosis risk has been further supported by large-scale validation studies, where proteins like C4B and C5 showed nominally significant associations with transition to psychosis (Byrne et al., 2024). Moreover, the clinical relevance of the complement system may extend beyond diagnosis, with preliminary evidence suggesting that specific C4 structural variants (e.g., C4AS) could be protective against suicidal ideation and attempts in schizophrenia patients, opening new avenues for risk assessment (Sokolowski et al., 2024). This multi-layered evidence strongly supports the complement cascade not just as a marker of ‘sickness behavior’ but as a core pathophysiological driver in a subset of patients.

Beyond synaptic pruning, the complement system interacts with other neuroimmune pathways, contributing to a state of chronic low-grade neuroinflammation. The elevation of pro-inflammatory cytokines (IL-6, TNF-α) and acute-phase proteins (CRP) in the blood of SCZ patients suggests a systemic inflammatory response (Lu et al., 2026; Eren et al., 2023). However, the link between these peripheral signals and brain pathology is thought to be mediated, at least in part, by dysfunction of the blood-brain barrier (BBB). Indeed, elevated levels of inflammatory proteins like C4-ana and IL-6 in the peripheral circulation may reflect increased BBB permeability, allowing peripheral immune signals to access the brain, and *vice versa*. This bidirectional crosstalk between the peripheral immune system and the central nervous system creates a vicious cycle, where peripheral inflammation exacerbates central neuroinflammatory processes and synaptic dysfunction, thereby contributing to the progression of the disease and the manifestation of symptoms.

Beyond causal inference, the SOMAscan platform has provided complementary insights into marker biology and clinical stratification. Engelke et al. (2022) distinguished disease-related state markers (NTRK2, CNTN1, ROBO2, PLXNC1), which normalize with antipsychotic treatment, from medication-specific signatures (NCAM1, TNFRSF17), which are downregulated by antipsychotics independently of disease status. Furthermore, a two-protein SOMAscan panel (PRL + MRC2) discriminated schizophrenia from healthy controls with AUC 0.93, demonstrating that even minimal proteomic signatures can achieve high diagnostic performance (Engelke et al., 2022). These findings complement MR-based causal evidence and highlight the utility of multi-platform approaches.

The potent effect of antipsychotic medications on the circulating proteome cannot be overstated. As shown in Table 2, many of the most frequently reported markers, such as PRL, AHSG, and certain apolipoproteins, are directly modulated by antipsychotic treatment. The study by Engelke et al. (2022) was pivotal in this regard, as it showed that while immune-related markers like MRC2 were elevated in drug-free patients, others, like the neurodevelopmental proteins, showed normalization upon treatment. This complex interplay means that a proteomic signature obtained from a medicated patient reflects a composite of disease state and drug response, complicating its use as a pure diagnostic tool but also offering a potential avenue for monitoring treatment efficacy.

In addition to the consolidated markers listed in Table 2, several other proteins have emerged as promising candidates from Mendelian randomization studies but are not yet covered by standard commercial proteomic panels. These include DNER (a neurodevelopmental receptor, identified as a high-priority causal target for SCZ with colocalization evidence (Chen et al., 2025)), CTSS (an immune-regulatory cathepsin S, identified in combined Olink + SOMAscan MR studies (Bhattacharyya et al., 2025)), TNFSF12 (a pro-inflammatory cytokine associated with MDD risk (Chen et al., 2025)), FGF23 (a fibroblast growth factor with protective associations in MDD (Chen et al., 2025)), BTN2A1 and BTN3A2 (immunoglobulin superfamily members implicated in cross-disorder psychiatric risk (Zhang et al., 2025)), and FES (a proto-oncogene tyrosine kinase with potential roles in synaptic signaling (Zhang et al., 2025)). While these proteins have strong genetic and statistical evidence for causality, their absence from widely used targeted MS panels (e.g., BAK270, PQ500) and, in some cases, even from affinity-based panels, represents a significant translational gap. This highlights the urgent need for assay development to enable clinical validation and eventual implementation of these novel biomarkers. Furthermore, proteins such as BDNF (reduced in SCZ, meta-analysis evidence (Lu et al., 2026)) and ANKRD12 (identified in SCZ vs. BD differential proteomics (Smirnova et al., 2019)) were not included in Table 2 due to limited cross-platform validation or lack of commercial assay coverage; however, they remain relevant as candidate markers for specific research questions and warrant further investigation in targeted validation studies.

A critical observation, however, is the significant translational gap for MS-based platforms: many of the most promising new markers—including DNER, CTSS, and specific complement components—are not yet covered by standard commercial «ready to use» kits like BAK270 or PQ500, highlighting a key area for future MS assay development. In contrast, affinity-based platforms such as Olink and SOMAscan already cover a broader range of immune and neurodevelopmental proteins, though their relative quantification and aptamer-/antibody-dependent specificity require careful interpretation when transitioning to clinical assays.

## 6 Discussion

### 6.1 Controversies, gaps, and the case for genetic-proteomic integration

Despite the growing consensus regarding the utility of blood-based biomarkers, several critical challenges remain that complicate the clinical application of proteomics in schizophrenia. First, the field is plagued by heterogeneity in study design, sample type, and analytical platforms (Rodrigues et al., 2022; Ignjatovic et al., 2019; Geyer et al., 2019; Greco et al., 2017). Furthermore, the reproducibility of proteomic findings is critically affected by pre-analytical and technical variables that are often underreported. Differences in sample type (plasma vs. serum), anticoagulants used (EDTA vs. citrate), handling protocols (time to centrifugation, freeze-thaw cycles), and batch effects during LC-MS/MS analysis can introduce substantial variability and confound biological signals (Rodrigues et al., 2022; Ignjatovic et al., 2019; Geyer et al., 2019; Greco et al., 2017). For instance, the meta-analysis by Rodrigues et al. (2022) demonstrated that only 20 proteins overlapped between plasma and serum studies, underscoring the profound impact of sample matrix on biomarker discovery. Similarly, affinity-based platforms like Olink and SOMAscan, while highly sensitive, are susceptible to antibody cross-reactivity and aptamer binding specificity issues that may vary across batches (Álvez et al., 2025; Engelke et al., 2022). The field would benefit from adopting standardized operating procedures, including the use of common reference materials and internal standards, as well as mandatory reporting of pre-analytical conditions, to improve cross-study comparability and facilitate clinical translation. There is an ongoing debate on whether MS-based or affinity-based platforms are better suited for clinical translation. Proponents of targeted MS (e.g., MRM) argue that absolute quantification and higher specificity are indispensable for the clinical validation of biomarkers (Carr et al., 2014; Kalinowski et al., 2021; Strelnikova et al., 2025; Lesur et al., 2023). At the same time, proponents of affine methods (Olink, SOMAscan) emphasize their superior performance and sensitivity in large-scale screening, which allows the search for cause-and-effect relationships using Mendelian randomization (Álvez et al., 2025; Engelke et al., 2022). A second controversy concerns the interpretation of elevated complement and inflammatory proteins: while some view them as core pathophysiological drivers of synaptic pruning and neuroinflammation (Woo et al., 2020; Nimgaonkar et al., 2017; Heurich et al., 2022; Sek et al., 2016; Fromer et al., 2016), others caution that they may largely reflect non-specific sequelae of antipsychotic treatment or lifestyle factors (Lu et al., 2026; Engelke et al., 2022). Another critical limitation is the relative lack of specificity of many candidate biomarkers for schizophrenia. Proteins such as CRP, IL-6, and TNF-α are established markers of systemic inflammation that are elevated across a wide range of psychiatric (e.g., major depressive disorder, bipolar disorder) and somatic (e.g., cardiovascular disease, autoimmune disorders) conditions (Lu et al., 2026; Eren et al., 2023; Malmqvist et al., 2025). This lack of diagnostic specificity does not diminish their potential utility—they may serve as valuable tools for monitoring disease activity, stratifying patients for anti-inflammatory interventions, or tracking treatment response. However, it underscores that no single protein marker is likely to function as a standalone diagnostic test for schizophrenia. Instead, the future of proteomic diagnostics lies in the development of multiplexed panels that combine multiple markers (e.g., inflammation, complement activation, synaptic proteins) to generate a composite risk or diagnostic score with improved specificity and clinical utility (Shin et al., 2024; Bhattacharyya et al., 2025). Third, the predictive value of proteomic signatures for CHR to psychosis transition remains disputed, as initial high optimism from the Mongan 2020 study (Mongan et al., 2020) was tempered by the large-scale validation study with much lower predictive accuracy (Byrne et al., 2024). This illustrates a broader field-wide tension between discovery-driven optimism and the necessity of rigorous, multi-cohort validation. Too many candidates are reported without rigorous independent validation in large, well-powered cohorts. A positive exception is the systematic use of the SOMAscan platform, which enabled both the distinction of medication effects from disease biology (Engelke et al., 2022) and the validation of causal markers through combined Olink + SOMAscan Mendelian randomization (Bhattacharyya et al., 2025). While Mendelian randomization has emerged as a powerful tool to infer causality from observational proteomic data, its limitations must be explicitly acknowledged. First, the validity of MR results hinges on the three core assumptions: (1) the genetic instrument is robustly associated with the protein, (2) it is not associated with confounders, and (3) it affects the outcome only through the protein (no horizontal pleiotropy). Violations of these assumptions, particularly pleiotropy, can lead to spurious causal claims. Second, the generalizability of MR findings is limited by the ancestral composition of the GWAS cohorts used (predominantly European populations in UK Biobank-based studies), which may not be applicable to other ethnic groups. Third, MR provides evidence for a causal link at the population level but does not directly inform us about the direction of causality in individual patients or the dynamic, state-dependent nature of protein changes. Therefore, MR results should be viewed as a hypothesis-generating filter that prioritizes proteins for functional validation, rather than definitive proof of a therapeutic target. Nevertheless, the field needs more such harmonized, multi-platform validation efforts. The detection of low-abundance proteins in plasma remains technically challenging, but targeted assays for activation products like C4-ana show promise (Kalinowski et al., 2021). Despite the growing body of evidence, it is crucial to temper expectations regarding the imminent clinical availability of these biomarkers. The field is currently in a phase of intensive discovery and initial validation; true clinical implementation requires overcoming substantial hurdles, including the lack of standardized pre-analytical protocols, high inter-cohort variability, and the frequent failure of candidate markers to replicate in independent, large-scale studies. Therefore, any discussion of ‘commercial panels’ must be framed as a long-term aspirational goal rather than a near-term reality.

Another gap is the insufficient integration of proteomic findings with existing genetic architecture. Although large-scale MR studies have identified dozens of causal proteins (Zhang et al., 2025; Chen et al., 2025; Bhattacharyya et al., 2025), most routine proteomic studies still report differentially expressed proteins without cross-referencing them against GWAS data. This represents a missed opportunity: overlaying proteomic hits with genetic risk loci (e.g., the complement C4 locus (Sek et al., 2016), or the ITIH3/ITIH4 region) can immediately prioritize candidates for further validation. To refine this process, it is recommended that future discovery studies routinely include a “genetic prior” filter, flagging proteins whose genes map to known SCZ risk loci.

A notable proof-of-concept comes from Kopylov et al. (2023), who combined quantitative proteomics, metabolomics, and GWAS data from the same schizophrenia cohort. Although their GWAS did not reach genome-wide significance due to limited sample size, the authors deliberately overlaid nominally associated loci (e.g., SNPs in *PTGS1*, *GABBR2*, *GRM8*, *SCARB1*, and *CUBN*) with differentially expressed proteins (including APOB, ITIH1, ITIH4, and complement components) and altered metabolites. This integrative strategy allowed them to reconstruct a multi-layer molecular network linking lipid transport, steroidogenesis, complement activation, and thyroid hormone dysregulation—pathways that are now being independently validated by large-scale MR studies (Zhang et al., 2025; Chen et al., 2025; Bhattacharyya et al., 2025). While this study was underpowered for definitive causal inference, it successfully demonstrated the value of cross-referencing proteomic hits with genetic data even in modestly sized cohorts.

The importance of such multi-omic integration is powerfully underscored by the largest recent cross-disorder GWAS, which analyzed 14 psychiatric disorders in over one million cases (Grotzinger et al., 2025). That study identified five latent genomic factors, with the Schizophrenia/Bipolar factor showing strongest enrichment for excitatory neuron biology—a finding that directly converges with proteomic data placing complement and synaptic proteins at the center of SCZ pathophysiology. Critically, the authors demonstrated that disorders clustering on the same genomic factor are largely indistinguishable at the level of individual genetic variants. This implies that differential diagnosis between SCZ and bipolar disorder—a key clinical challenge—may ultimately require protein-level readouts rather than genetic testing alone. The polygenic architecture described by Grotzinger et al. (2025) thus provides a strong rationale for blood proteomics as an essential complementary layer that captures dynamic, state-dependent biology which genetics cannot reveal.

This integrative framework is schematically summarized in Figure 1, which illustrates the convergence of plasma proteomic platforms (mass spectrometry and affinity-based approaches) with genetic data (GWAS and Mendelian randomization) through multi-cohort validation, ultimately enabling precision psychiatry applications including early risk assessment, differential diagnosis, patient stratification, and targeted therapy.

**FIGURE 1:**
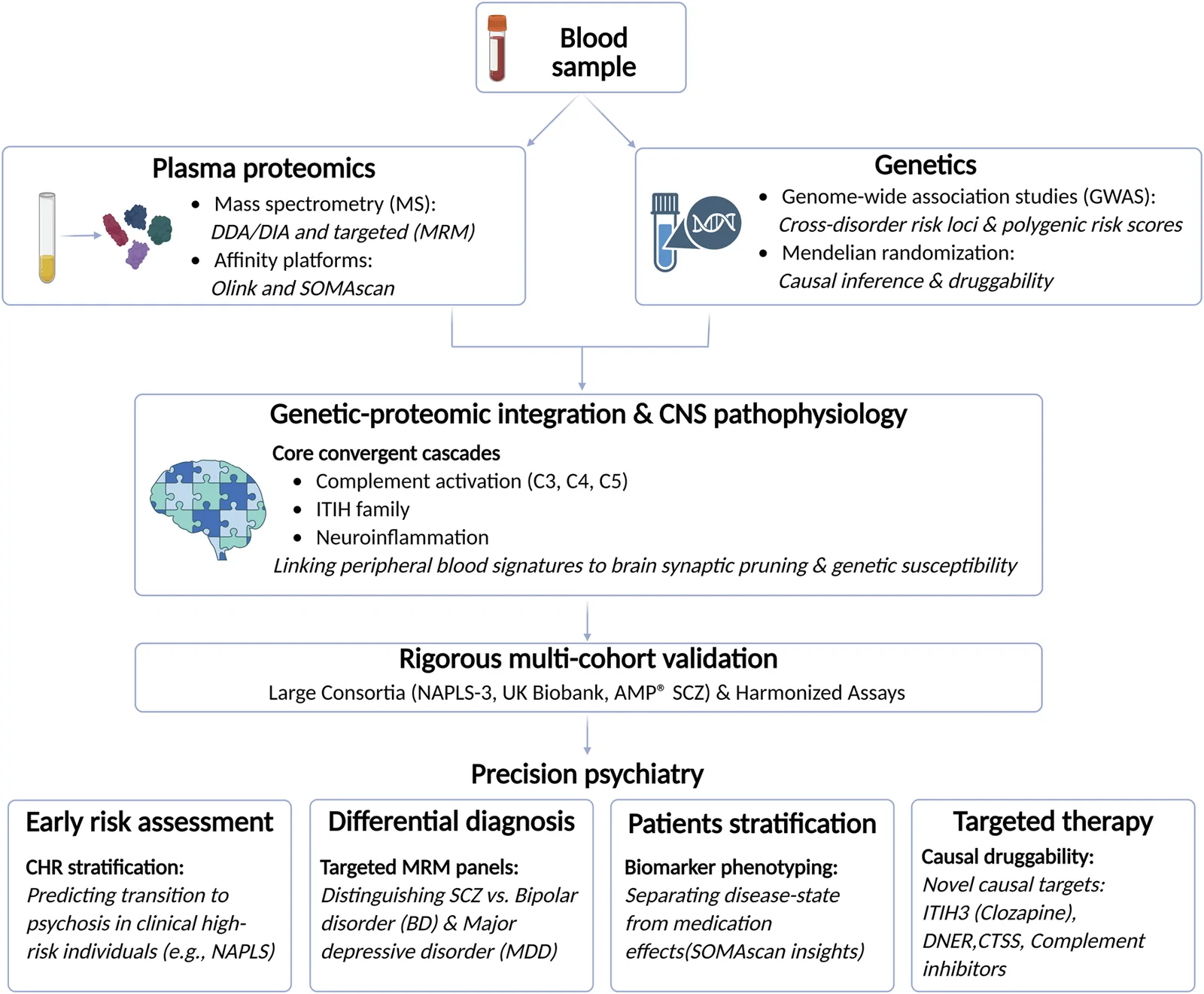
Integrative framework for precision psychiatry in schizophrenia through genetic-proteomic convergence. Created in BioRender. Borisov, D. (2026) https://BioRender.com/3lfvtrh.

### 6.2 Further research and implementation

Blood-based protein panels could fundamentally reshape psychiatric practice by enabling patient stratification for early intervention (Mongan et al., 2020; Byrne et al., 2024), guiding treatment selection (e.g., anti-inflammatory therapy for patients with elevated IL-6 or complement activation) (Lu et al., 2026; Eren et al., 2023; Woo et al., 2020; Nimgaonkar et al., 2017; Heurich et al., 2022), and monitoring treatment response and side effects (Lu et al., 2026; Engelke et al., 2022; Sokolowski et al., 2024). The definitive endpoint is the creation of validated, multiplexed biomarker panels that inform clinical decision-making. The work by Shin et al. (2024) proves the concept is feasible for differential diagnosis. The necessary evolution of the field involves a progression from initial, promising discovery to rigorous, large-scale validation that provides essential insights into generalizability and true effect sizes. This progression from discovery to large-scale validation is exactly the model the field needs to adopt, as exemplified by studies leveraging the infrastructure of major consortia like NAPLS-3 (Byrne et al., 2024; Addington et al., 2020), or initiatives like the Accelerating Medicines Partnership® Schizophrenia (AMP® SCZ) program which not only validate proteomic candidates but also integrate them with other biomarker classes to predict patients’ outcomes (Perkins et al., 2025).

In the next five to 10 years, we may see the emergence of the first ‘proof-of-concept’ commercial multiplexed proteomic tests for research use or for highly specific, niche clinical applications, with differential diagnosis being the most likely initial indication (Shin et al., 2024). However, widespread clinical adoption will critically depend on the outcomes of rigorous, multi-cohort validation studies currently underway, such as those within the AMP® SCZ program (Perkins et al., 2025). Its use for risk prediction in CHR individuals will also be informed by the results of large-scale validation studies (Byrne et al., 2024; Addington et al., 2020) and future initiatives designed to integrate multi-omic data. The complement system exemplifies the power of a multi-omic approach, where genetic variation in C4 confers risk (Sek et al., 2016), brain transcriptomics reveals elevated C4A expression (Fromer et al., 2016), and plasma proteomics detects increased C4 activation products (Kalinowski et al., 2021), while MR confirms causality (Bhattacharyya et al., 2025). Such convergent evidence across omic layers provides a level of biological plausibility that no single technology can achieve alone. Future research should prioritize multi-cohort studies that simultaneously collect genomic, proteomic (both MS- and affinity-based), and transcriptomic data from the same individuals, following the template of initiatives like AMP® SCZ (Perkins et al., 2025). The ultimate goal is a multi-omic risk score that integrates protein, genetic, and clinical data to predict not only the diagnosis but also treatment response and long-term outcomes.

This area is the most promising avenue for advancing precision psychiatry and understanding the immune-metabolic-synaptic axis, with the complement system playing a central role. Markers like ITIH3 (dopamine modulation and immune response), DNER (neurodevelopment), and C4 (synaptic pruning) represent entirely new classes of intervention points (Lu et al., 2026; Zhang et al., 2025; Chen et al., 2025; Woo et al., 2020).

## 7 Conclusion

Despite significant progress, a clinically validated blood biomarker panel for schizophrenia does not yet exist. Panoramic proteomic studies have generated numerous hypotheses but suffer from poor reproducibility. However, the field is maturing. Targeted MS has demonstrated high diagnostic accuracy in differential diagnosis, while large-scale Mendelian randomization studies have identified potentially causal proteins, many with druggable potential. Affinity-based platforms have further enabled the distinction of disease-related state markers from medication effects.

Critically, recent cross-disorder GWAS has shown that genetic architecture alone cannot reliably distinguish schizophrenia from bipolar disorder at the individual variant level, underscoring the need for protein-level readouts that capture dynamic, state-dependent biology. This convergence of genetic and proteomic evidence provides a strong rationale for integrating both layers in future biomarker development.

The path forward requires expanding commercial panels to include emerging markers such as DNER, CTSS, and specific complement activation products, alongside rigorous multi-cohort validation following the models of NAPLS-3 and AMP® SCZ. Moving beyond the discovery phase, the field is now entering a stage of clinical translation driven by the synergy between affinity-based platforms and targeted mass spectrometry. Ultimately, proteomic and multi-omic stratification will facilitate the identification of biologically distinct patient subgroups, anchoring psychiatry within a robust precision medicine framework.
